# The large fraction of heterochromatin in *Drosophila* neurons is bound by both B-type lamin and HP1a

**DOI:** 10.1186/s13072-018-0235-8

**Published:** 2018-11-01

**Authors:** Alexey V. Pindyurin, Artem A. Ilyin, Anton V. Ivankin, Mikhail V. Tselebrovsky, Valentina V. Nenasheva, Elena A. Mikhaleva, Ludo Pagie, Bas van Steensel, Yuri Y. Shevelyov

**Affiliations:** 1 Division of Gene Regulation, Netherlands Cancer Institute, 1066 CX Amsterdam, The Netherlands; 2 Department of Regulation of Genetic Processes, Institute of Molecular and Cellular Biology, Siberian Branch of Russian Academy of Sciences, Novosibirsk, Russia 630090; 3 Laboratory of Structural, Functional and Comparative Genomics, Novosibirsk State University, Novosibirsk, Russia 630090; 4 Department of Molecular Genetics of Cell, Institute of Molecular Genetics, Russian Academy of Sciences, Moscow, Russia 123182; 5 Present Address: QC Biochemistry Lab, Yaroslavl Pharmaceutical Complex for Production of Finished Dosage Forms, R-Pharm Group, Yaroslavl, Russia 150061; 6 Department of Viral and Cellular Molecular Genetics, Institute of Molecular Genetics, Russian Academy of Sciences, Moscow, Russia 123182; 7 Department of Cell Biology, Erasmus University Medical Center, 3015 GE Rotterdam, The Netherlands

**Keywords:** Heterochromatin, HP1, B-type lamin, Lamina-associated domains, Polycomb, *Drosophila*

## Abstract

**Background:**

In most mammalian cell lines, chromatin located at the nuclear periphery is represented by condensed heterochromatin, as evidenced by microscopy observations and DamID mapping of lamina-associated domains (LADs) enriched in dimethylated Lys9 of histone H3 (H3K9me2). However, in Kc167 cell culture, the only *Drosophilla* cell type where LADs have previously been mapped, they are neither H3K9me2-enriched nor overlapped with the domains of heterochromatin protein 1a (HP1a).

**Results:**

Here, using cell type-specific DamID we mapped genome-wide LADs, HP1a and Polycomb (Pc) domains from the central brain, Repo-positive glia, Elav-positive neurons and the fat body of *Drosophila* third instar larvae. Strikingly, contrary to Kc167 cells of embryonic origin, in neurons and, to a lesser extent, in glia and the fat body, HP1a domains appear to overlap strongly with LADs in both the chromosome arms and pericentromeric regions. Accordingly, centromeres reside closer to the nuclear lamina in neurons than in Kc167 cells. As expected, active gene promoters are mostly not present in LADs, HP1a and Pc domains. These domains are occupied by silent or weakly expressed genes with genes residing in the HP1a-bound LADs expressed at the lowest level.

**Conclusions:**

In various differentiated *Drosophila* cell types, we discovered the existence of peripheral heterochromatin, similar to that observed in mammals. Our findings support the model that peripheral heterochromatin matures enhancing the repression of unwanted genes as cells terminally differentiate.

**Electronic supplementary material:**

The online version of this article (10.1186/s13072-018-0235-8) contains supplementary material, which is available to authorized users.

## Background

Eukaryotic chromosomes are subdivided into less condensed euchromatin and more densely packed heterochromatin. The facultative heterochromatin that is dispersed on the chromosome arms (hereafter ChAs) is mostly composed of silent tissue-specific genes and transposable elements (TEs), whereas pericentromeric and telomeric regions highly enriched in satellite DNA, TEs and other repeats form the constitutive heterochromatin (the 2LHet, 2RHet, 3LHet, 3RHet, XHet chromosome regions of dm3/R5 genome assembly; hereafter CHet) (reviewed in [1, 2]). Immunostaining and electron microscopy observations indicate that in mammalian cells, both the facultative and constitutive heterochromatin are located close to the nuclear envelope and around the nucleoli, with an interesting exception being the rod photoreceptor cells of animals with nocturnal vision, where the heterochromatin is centrally positioned ([3] and references therein).

The nuclear envelope is lined with A- and B-type lamin filaments which, together with numerous lamin-binding proteins, compose the nuclear lamina (reviewed in [4, 5]). Using the DamID approach [6, 7], lamina-associated chromosomal domains (LADs) were revealed in *Drosophila*, nematode and mammalian cell lines [8–13]. LADs mostly harbor silent or weakly expressed genes [9, 11, 12]. Accordingly, the nuclear lamina was shown to be a repressive environment for transcription [14–22]. In mammals, LADs correspond to chromatin domains enriched with the dimethylated Lys9 of histone H3 (H3K9me2) mark [9, 23–25], whereas the trimethylated Lys27 of histone H3 (H3K27me3) mark is enriched at the LAD borders [9]. The H3K9me2-modified nucleosomes may be bound by the heterochromatin protein 1a (HP1a) [26–28], and the H3K27me3 mark may recruit the Polycomb group (PcG) proteins [29–32]. Binding of both repressors condenses chromatin [33–38], thus forming the adjoining nuclear lamina heterochromatin layer (reviewed in [39]). However, in *Drosophila*, LADs have previously only been mapped in cultured Kc167 cells of embryonic origin [11], where they are enriched neither in H3K9me2 nor in HP1a [40]. Moreover, less than half of LADs in Kc167 cells are enriched in Polycomb (Pc) binding [11]. This raises the question of whether the heterochromatin located at the nuclear periphery in other *Drosophila* cell types may be bound by HP1a or, to a greater extent, by Pc.

Recent modifications of the DamID technique have made it possible to map the interactions of proteins of interest (POIs) with chromatin in a particular cell type within complex tissues [41–46]. Using such an approach, the chromosomal regions interacting with the Pc repressor in the fat bodies, the whole central brain and Repo-positive glial cells of the central brain of *Drosophila* third instar larvae were previously mapped genome wide [44]. In this study, to map the landscape of repressive chromatin types more comprehensively, we also mapped HP1a and the B-type lamin Dm0 (hereafter Lam) in the same organs/cell types. Furthermore, we mapped interactions with Pc, HP1a and Lam in the Elav-positive neurons of the central brain. In neurons and, to a lesser extent, in glia and fat bodies, we found that a substantial portion of heterochromatin interacts with both Lam and HP1a. Importantly, such a specific composition of heterochromatin has not been previously described for *Drosophila*. Finally, we revealed that centromeres are positioned closer to the nuclear lamina in *Drosophila* neurons than in Kc167 cells.

## Results

### DamID mapping of Pc, Lam and HP1a domains in various cell types of *Drosophila* larvae

**Fig. 1:**
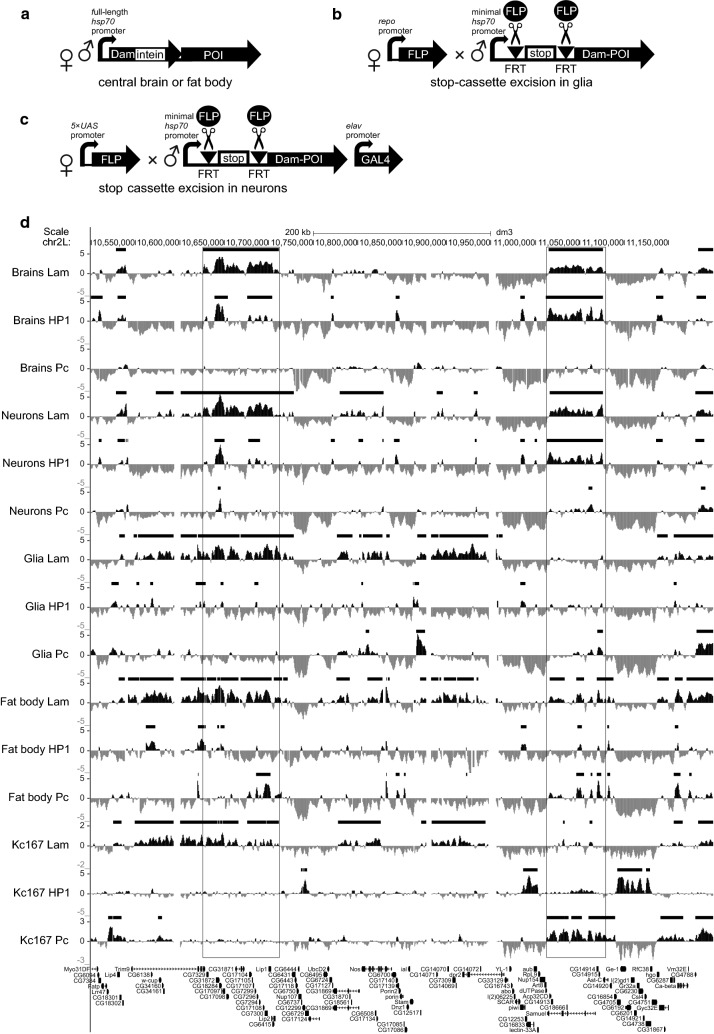
DamID mapping of LADs, HP1a and Pc domains in the central brain, neurons, glia and fat body. **a**–**c** Principles of DamID in the central brain and fat body (**a**), glia (**b**) and neurons (**c**). POI is Lam, HP1a or Pc. **d** Screenshot from UCSC genome browser showing log_2_(Dam-POI/Dam) profiles and HMM-determined domains of POI enrichment (black rectangles over profiles) for the representative region of 2L chromosome in the central brain, neurons, glia, fat body and Kc167 cells. Data for Pc in all organs/cell types except neurons are taken from [44]. Data for Kc167 cells are taken from [11, 40]. Examples of LADs completely or partially overlapped with HP1a domains in the central brain or neurons but not in glia, fat body and Kc167 cells are outlined by black rectangles

DamID-seq profiles of genome-wide Pc binding from the larval central brain, Repo-positive glial cells and fat body cells have been reported previously [44]. The corresponding profiles of HP1a and Lam were generated at the same time; thus, they all share the same Dam only normalization controls (Fig. 1a, b). DamID-seq profiles of POIs (Pc, Lam and HP1a) in neurons were obtained by using the FLP-inducible STOP#1-Dam system [44] combined with the pan-neuronal *elav*-*GAL4* driver and a *UAS*-*FLP* transgene (Fig. 1c, Additional file 1). Amplification of Dam-methylated fragments of the neuronal genome was performed as previously described for glial cells [45]. The high specificity of the amplification procedure was confirmed by gel electrophoresis showing substantially more mePCR products in experimental samples compared to negative controls, in which STOP#1 DamID transgenes were not activated by GAL4 protein (Additional file 2: Fig. S1). Subsequent high-throughput sequencing (HTS) of these mePCR samples was performed according to [44].

Next, unique mapping of sequence reads of all studied DamID-seq samples to 1-kb bins of the *Drosophila* dm3/R5 genome assembly was performed. This resulted in a high correlation between replicates of Dam-POI and Dam genome-wide binding profiles (Additional file 2: Fig. S2; for Pc mapping in the central brain, fat body and Repo-positive glial cells we employed previously obtained data from GSE75835 [44]). Then, replicates were merged, and the resulting Dam-POI profiles were normalized to the corresponding Dam profiles and log_2_ transformed. After that, for each POI, the quantile normalization between organs/cell types was applied. Finally, the chromatin domains enriched for Pc, Lam and HP1a interactions were determined for each organ/cell type using the hidden Markov model (HMM) algorithm (Fig. 1d, Additional file 2: Fig. S3, Additional file 3: Table S1). For further bioinformatic analysis, we additionally employed domain enrichment data for the Pc, Lam and HP1a in embryonic Kc167 cell culture reported previously [11, 40].

### Pc, Lam and HP1a domains are not conserved among different *Drosophila* cell types

**Fig. 2:**
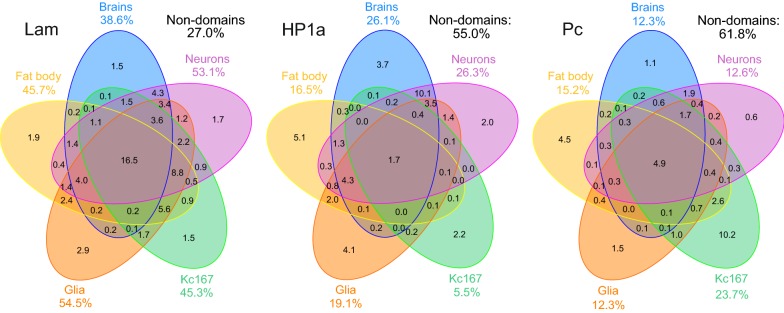
Common LADs, HP1a and Pc domains in various cell types. Diagrams showing the degree of overlap (as a percentage of ChAs length) between (left to right) LADs, HP1a or Pc domains in the central brain, neurons, glia, fat body and Kc167 cells

Depending on cell type, chromosomal regions interacting with Lam (i.e., LADs) in *Drosophila* occupy from 39% (in the central brain) to 55% (in glia) of the length of ChAs, whereas HP1a domains cover 6–26% and Pc domains—12–24% of ChAs (Fig. 2). Minimal LAD coverage in the central brain likely reflects the brains composition of different cell types. Therefore, similar to mammals [10], LADs represent the most prominent type of inactive chromatin domains in *Drosophila*. However, unlike in mammals, where the conserved LADs comprise about 33% of non-repetitive genome [47], the LADs shared among various *Drosophila* cell types were less abundant (occupying 16.5% of ChAs, Fig. 2). The conserved HP1a and Pc domains span the minor part of ChAs (1.7% and 4.9%, respectively). Importantly, in the analyzed cell types the shared inter-domains (i.e., the regions which do not significantly interact with the corresponding POI) were remarkably represented for each of these repressors. For example, 27% of the *Drosophila* genome does not typically interact with the nuclear lamina in any of the cell types analyzed (Fig. 2). This value is 1.4-fold less than observed in mice (38% [47]). Therefore, the variability of LADs in *Drosophila* is much higher than in mammals.

### HP1a domains highly overlap with LADs in neurons, but not in Kc167 cells

**Fig. 3:**
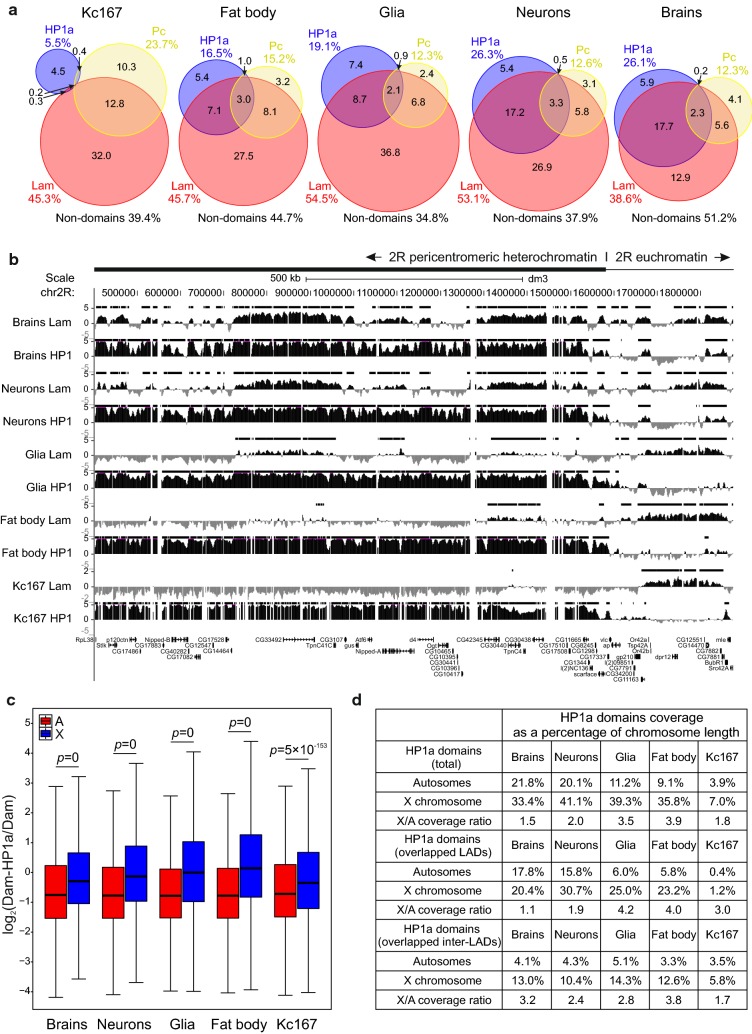
LADs strongly overlap with HP1a domains (both in the ChAs and in the pericentromeric regions) in the central brain and neurons, to a lesser extent in glia and the fat body, and not at all in Kc167 cells. **a** Venn diagram showing the degree of overlap as a percentage of ChAs length between LADs, HP1a and Pc domains (left to right) in Kc167 cells, fat body, glia, neurons or the central brain. **b** Screenshot from UCSC genome browser showing log_2_(Dam-POI/Dam) profiles (where POI is Lam or HP1a) and HMM-determined domains (black rectangles over profiles) for the representative 2R pericentromeric region in the central brain, neurons, glia, fat body and Kc167 cells. Data for Kc167 cells were taken from [11, 40]. The eu/heterochromatin boundary (thick/thin black line above the figure) is indicated according to [64]. **c** Box plots showing distributions of log_2_(Dam-HP1a/Dam) values in the non-repetitive parts of X chromosome (blue) and autosomes (red) in male larval central brain, male larval fat bodies, neurons or glial cells from mixed sex larvae, and in the female Kc167 cells. For this type of analysis, raw DamID-seq data for HP1a in Kc167 cells were taken from GSE83713 [67], mapped on the 1-kb genomic bins and quantile normalized. M–W *U* test was used for pairwise comparison of distributions on the X chromosome *vs* autosomes. **d** HP1a domain coverage on the X chromosome and autosomes as a percentage of chromosomes length. Only the ChA parts which, according to Riddle et al. [64], were within 1–22,300 kb for X chromosome, 1–22,000 kb for 2L, 1600–21,147 kb for 2R, 1–22,900 kb for 3L, 1–27,900 kb for 3R of *Drosophila* dm3/R5 genome assembly, were taken for analysis

We further analyzed the degree of overlap between different domain types. Consistent with the results of Filion et al. [40], LADs and HP1a domains appear to overlap very poorly in the cultured Kc167 cells (the overlapped regions constitute 10% of the length of HP1a domains, 1% of LADs length and 0.5% of ChAs length; Fig. 3a, Additional file 4: Table S2). However, in various larval organs/cell types the degree of overlap between these two domain types was notably higher, with the major intersection of LADs and HP1a domains occurring mainly in the central brain and in neurons (in the central brain and neurons, the overlap constitutes 77–78% of total HP1a domain length, 38–52% of total LADs length and covers ~ 20% of ChAs; Figs. 1d, 3a, Additional file 4: Table S2). The observed genome distributions of LADs and HP1a domains are highly non-random (in each case *p *< 10^−4^, permutation test). Moreover, the increased overlap between LADs and HP1a domains in the analyzed organs/cell types relative to Kc167 cells is characteristic not only for ChAs but also for the pericentromeric regions (Fig. 3b), where the degree of overlap is even higher (HP1a/LADs intersection length varies from 68% (in fat body) to 91% (in the central brain) of total HP1a domain length in CHet; Additional file 4: Table S2). We note that the pattern of LADs/HP1a overlap for the 4th chromosome is different from that in ChAs and CHet (Additional file 2: Fig. S4, Additional file 4: Table S2). In contrast to varying overlap between LADs and HP1a domains, the degree of intersection between LADs and Pc domains was more similar in different organs/cell types analyzed (the overlap constitutes 17–29% of LADs, 55–73% of Pc domains and covers 8–13% of ChAs; Fig. 3a, Additional file 2: Fig. S5, Additional file 4: Table S2). These results uncover the interactions of chromatin with both the nuclear lamina and HP1a, which are most prominent in *Drosophila* neurons.

To test the hypothesis that HP1a binding in LADs may be mediated by TEs, we analyzed the distribution of 3183 TE insertions in the ChAs of *Drosophila* reference genome. We found that in the central brain, TE occupancy in the close vicinity of HP1a-bound LADs is twofold higher (*p *< 10^−4^, permutation test) than that in LADs without HP1a binding, where it appears to be the same as in the whole ChAs. We, thus, propose that TEs in the central brain may participate in the recruitment of HP1a to LADs.

Previously, the preferential binding of HP1a with the X chromosome as compared to autosomes was revealed in adult *Drosophila* males, but not in females [48]. In agreement with these results, the profile of HP1a in the non-repetitive part of X ChA is shifted toward higher values when compared to autosomes in the male larval central brain and fat body, as well as in neurons and glial cells isolated from a mixed population of both sexes (Fig. 3c). This X chromosome-specific HP1a enrichment is lower (but still present) in female Kc167 cells (Fig. 3c). Interestingly, HP1a is bound to a larger number of sites on the X chromosome as compared to autosomes (both in LADs and in the inter-LADs) in all organs/cell types analyzed (Fig. 3d). In the central brain, HP1a domains overlapping with LADs cover similar genome fractions on the X chromosome and on autosomes, yet the increased HP1a binding on the X chromosome relative to autosomes is revealed (Additional file 2: Fig. S6). These results are consistent with the generally elevated HP1a binding with the X chromosome, which is especially evident in males.

### The expression level of genes in LADs, Pc and HP1a domains is generally very low

**Fig. 4:**
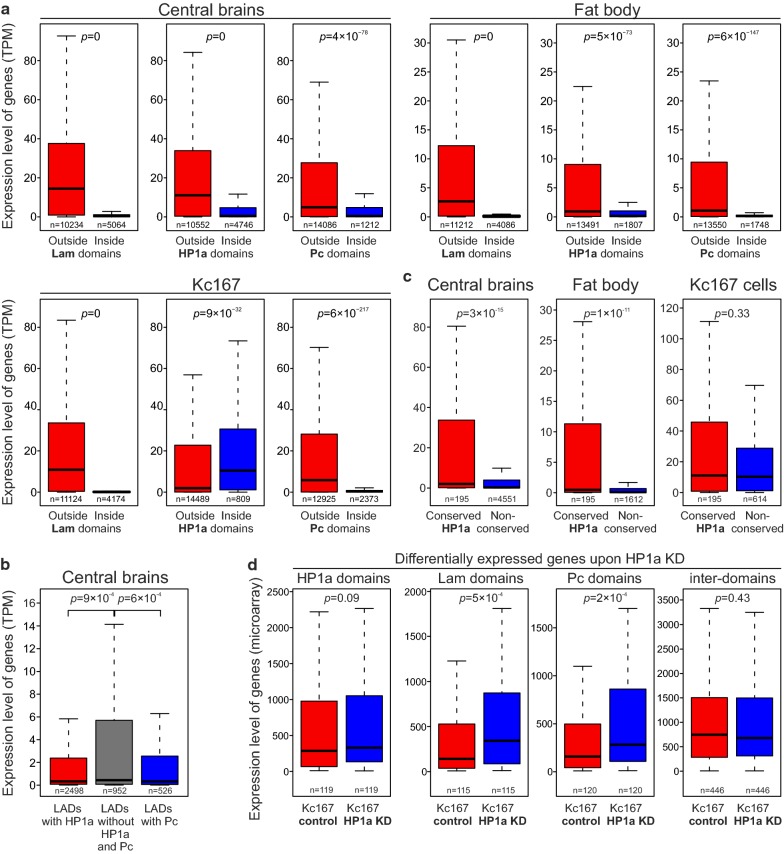
Expression level of genes residing in LADs, Pc and HP1a domains is drastically lower than in the inter-domains in the central brain and fat body, except for the HP1a domains in Kc167 cells. **a** Box plots (non-outlier range) showing expression of genes (in TPM) overlapping (blue) or non-overlapping (red) by their promoters (distal TSSs) with LADs, HP1a and Pc domains in larval central brain, fat body and Kc167 cells. RNA-seq data for larval central brain and fat body were taken from GSE75835 [44], and for Kc167 cells—from GSE15596 [49]. **b** Box plots (non-outlier range) for the central brain showing the expression of genes (in TPM) whose promoters (distal TSSs) are located in LADs, (left to right) overlapped with HP1a domains (red), non-overlapped with either HP1a or Pc domains (gray), or overlapped with Pc domains (blue). Zero TMP values were excluded from the analysis. **c** Box plots (non-outlier range) showing expression levels of genes (in TPM) overlapped with the conserved HP1a domains by their promoters (distal TSSs) in the central brain, fat body and Kc167 cells. **d** Box plots (non-outlier range) showing expression levels for the differentially expressed genes, whose bodies overlap with HP1a, Lam or Pc domains, or those not overlapped with any domain type, in the control Kc167 cells (red) or upon HP1a KD in Kc167 cells (blue). RNA expression microarray data for analysis were taken from GSE18092 [50]. In (**a**–**d**), M–W *U* test was used for pairwise comparison of distributions

To analyze the expression levels of genes residing in LADs, Pc and HP1a domains, we employed previously obtained RNA-seq data for the central brain and fat bodies isolated from the third instar larvae males (GSE75835 [44]), or for Kc167 cells (GSE15596 [49]). In the central brain and fat body, we found drastically lower expression levels of genes whose promoters (distal TSSs) are located in LADs, Pc and HP1a domains, as compared to the inter-domains (Fig. 4a, Additional file 5: Table S3). The same picture is seen in Kc167 cells, but only for the genes whose promoters reside in LADs and Pc domains (Fig. 4a). Moreover, in the central brain, the expression level of genes, whose promoters are found in LADs and are simultaneously bound by HP1a or Pc, appears to be lower than in LADs lacking these proteins (Fig. 4b). A similar trend is revealed in the fat body for the promoters residing in LADs overlapped with the HP1a domains (Additional file 2: Fig. S7A). These results support the model that HP1a or Pc binding introduces an additional layer of gene repression in LADs.

However, in Kc167 cells, median expression level of genes, whose promoters (distal TSSs) are located in the HP1a domains, appears to be increased compared to the rest of the ChAs (Fig. 4a), therefore indicating the interaction of HP1a with the actively expressed genes in these cells. The difference is even more pronounced for the genes overlapping with HP1a domains by their bodies (Additional file 2: Fig. S7B). Because approximately one third of HP1a domains in Kc167 cells are preserved in other organs/cell types (Fig. 2, Additional file 2: Fig. S8), we examined in the central brain and fat body the expression level of genes located in these conserved HP1a domains. As expected, gene expression appears to be notably higher in the conserved than in the non-conserved HP1a domains in these organs, whereas in Kc167 cells it is rather similar (Fig. 4c). Therefore, a fraction of HP1a is bound to the actively expressed genes not only in Kc167 cells, but also in the other analyzed cell types.

In attempts to clarify the effect of HP1a on those genes, we used publicly available microarray expression data for the control and HP1a-depleted Kc167 cells from GSE18092 [50]. Out of 707 differentially expressed genes in the control and HP1a-depleted cells (Additional file 6: Table S4), 119 genes overlap with the HP1a domains by their bodies, therefore being the direct HP1a targets. As expected, these genes are actively expressed in Kc167 cells (Additional file 2: Fig. S7C). Upon HP1a knockdown (KD), 74 out of 119 genes were up- and 45 genes were down-regulated; however, the median expression of these HP1a targets was not notably changed (Fig. 4d). At the same time, the differentially expressed genes, whose bodies overlap with LADs or Pc domains, were significantly up-regulated upon HP1a depletion (Fig. 4d). The same results were obtained when only promoters (distal TSSs) residing in the corresponding domains were considered (not shown). Next, we analyzed the differentially expressed genes with more than twofold expression difference upon HP1a KD and revealed the profound up-regulation of direct HP1a targets (Additional file 2: Fig. S7D). However, it was accompanied by the increased expression of genes residing in LADs, in Pc domains and those outside of any domain type (Additional file 2: Fig. S7D). We note that among 1647 genes that overlap with the HP1a domains by their bodies in Kc167 cells, only ~ 7% have significantly altered expression after HP1a KD. Altogether, this pointed to the mild (if any) effect of HP1a on the transcription or RNA stability of its actively expressed direct targets, and on the presence of indirect effect of HP1a depletion on the transcription.

### Active promoters avoid association with the nuclear lamina or Pc

**Fig. 5:**
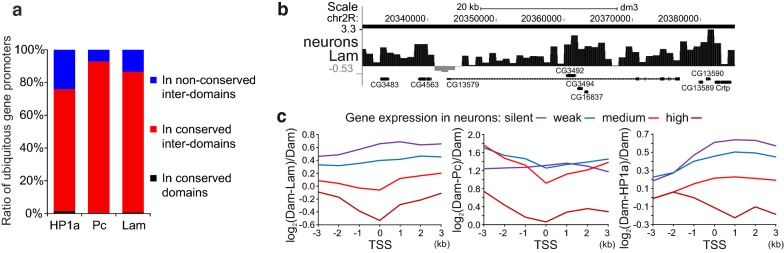
Actively expressed gene promoters are not associated with the nuclear lamina and Pc. **a** Promoters (distal TSSs) of ubiquitously expressed genes are mainly localized in the conserved HP1a, Lam or Pc inter-domains. The percentage of ubiquitously expressed gene promoters residing in the non-conserved inter-domains (blue), conserved inter-domains (red) or conserved domains (black) is indicated. Regions common to the central brain, neurons, glia, fat body and Kc167 cells are operationally determined as conserved. **b** Screenshot from UCSC genome browser showing an example of the gene *CG13579*, which is specifically expressed in neurons, with the dip of the neuronal log_2_(Dam-Lam/Dam) profile at the TSS region. Note that due to imperfectness of domain calling algorithm no gap in the HMM-determined Lam domain (black rectangle over profile) is seen. **c** Plotted are the median values of neuronal log_2_(Dam-POI/Dam) profiles (where POI is Lam, Pc or HP1a) around promoters (distal TSSs) of the genes separated according to their neuronal expression level into four groups

Because LADs mostly correspond to the silent genome regions ([9, 11], these data), we hypothesized that conserved inter-LADs may be populated specifically by the ubiquitously expressed genes which are active in any cell type. To examine this possibility, we generated a list of 4377 ubiquitously expressed genes (Additional file 7: Table S5) by the criterion that their expression should exceed background in any of *Drosophila* tissues represented in the FlyAtlas database [51]. Our analysis indicates that the vast majority of ubiquitously expressed gene promoters (distal TSSs) are indeed localized in the common for all cell types Lam, Pc or HP1a inter-domains (86%, 93% and 75%, respectively; Fig. 5a), covering 27%, 62% or 55% of ChAs (Fig. 2). These localization patterns are highly non-random (*p* < 10^−4^ for each POI, permutation test). Yet, a small fraction of those promoters (14%, 7% or 25%) are intersected with LADs, Pc or HP1a domains in at least one cell type, and only the minor fraction of them overlaps with the LADs, Pc or HP1a domains conserved among various cell types (Fig. 5a). Therefore, promoters of ubiquitously expressed genes are almost always located outside LADs, Pc domains and, to a lesser degree, outside the HP1a domains.

We next analyzed whether the tissue-specifically expressed gene promoters are localized in the inter-domains. Based on the RNA-seq data from [52], we generated a list of tissue-specific genes expressed in the larval neurons or glial cells. The major fraction of promoters of these genes appears to locate in the inter-domains (64% or 61% TSSs fall in the inter-LADs, 74% or 67% fall in the HP1a inter-domains and 93% or 92% fall in the Pc inter-domains in neurons or glia, respectively) which is significantly deviated from the random distribution (*p* < 10^−4^ for each POI, permutation test). Upon examination of DamID profiles, we noticed that some promoters of tissue-specifically expressed genes lose their interactions with the nuclear lamina, whereas their bodies stay in contact. For example, the log_2_(Dam-Lam/Dam) profile in neurons has a dip at the promoter region of neuron-specific *CG13579* gene, whereas the elevated association with the nuclear lamina is revealed along its body (Fig. 5b). This dip is absent in the profile for glial cells (not shown), where *CG13579* is not expressed. We separated all the genes into four groups according to their expression level and plotted medians of log_2_(Dam-POI/Dam) values around TSSs for the genes aligned in the 5′ → 3′ direction and overlapped by their bodies with the corresponding domain type. We found that the higher genes are expressed—the weaker the Lam, Pc or HP1a binding at their promoters (Fig. 5c). Moreover, the local minima values in the Lam, or Pc profiles, plotted for the genes with the medium or high expression, fall into the 1-kb bin containing TSSs. A similar picture is seen for the glial profiles (not shown). Interestingly, the HP1a profiles for the actively expressed genes in both neurons (Fig. 5c, right panel) and glia (not shown), unlike Lam or Pc profiles, display the local minima values in the gene bodies, but not at the TSSs. We conclude that, as in mammals [10, 53, 54], promoters of a small fraction of actively expressed genes located in *Drosophila* LADs are mostly released from an association with the nuclear lamina. The same trend is seen for the genes residing in the Pc domains.

### Centromeres are located closer to the nuclear lamina in neurons than in Kc167 cells

**Fig. 6:**
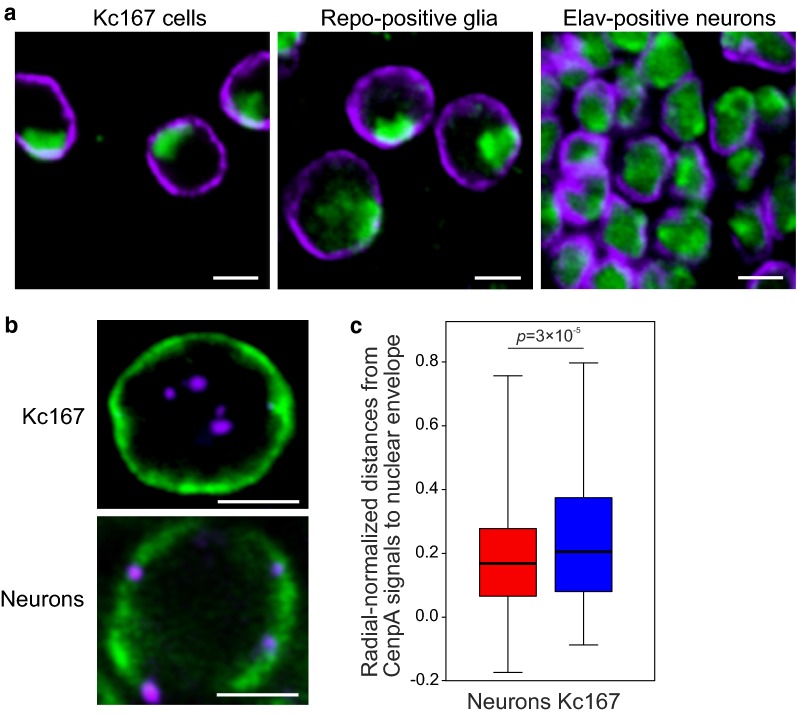
HP1a is more evenly distributed in the nucleus, and centromeres are located more closely to the nuclear envelope in neurons than in Kc167 cells. **a** Immunostaining of Kc167 cells (left panel), Repo-positive glia (central panel) and Elav-positive neurons (right panel) with anti-HP1a (green) and anti-Lam (violet) antibodies. Scale bars 3 μm. **b** Confocal images of immunostaining of Kc167 cells (upper panel) or Elav-positive neurons (lower panel) with anti-CenpA (violet, a marker of centromeres) and anti-Lam (green) antibodies. Shown are the examples of nuclei with the centromeres located at the nuclear envelope in neurons or clustered in the nuclear interior in Kc167 cells. Scale bars 3 μm on the upper panel or 2 μm on the lower panel. **c** Box plots showing radial-normalized distances between CenpA signals and the nuclear lamina in neurons (red bars, n = 468) and Kc167 cells (blue bars, n = 604), counted in 3D using IMARIS software. In cases when the centers of CenpA signals were shifted toward the outer side of the nuclear lamina relative to its midpoint, negative distances were assigned. M–W *U* test was used for pairwise comparison of two distributions

Our DamID results demonstrate the strong overlap of LADs and HP1a domains in neurons and almost complete lack of such an overlap in Kc167 cells. To better understand this phenomenon, we immunostained neurons, glia and Kc167 cells with anti-HP1a and anti-Lam antibodies and examined the distribution of HP1a in the nucleus. Neuronal or glial nuclei in the third instar larvae brain were marked by the fluorescence of DsRed.T4 protein in *elav*-*GAL4* × *UAS*-*RedStinger* or *repo*-*GAL4* × *UAS*-*RedStinger* crossed flies, respectively. In agreement with the previously published HP1a distribution in Kc167 cells [55], we found that in Kc167 or glial cells, HP1a occupies 1–2 clearly stained pericentromeric compartments (Fig. 6a), whereas in neurons it is more uniformly distributed in the nucleus, probably reflecting more abundant binding of HP1a to the ChAs in the latter case.

Next, we examined whether the pericentromeric regions are positioned closer to the nuclear lamina in neurons than in Kc167 cells. We immunostained interphase nuclei of both cell types with anti-CenpA (CID) antibodies marking the centromeres [56, 57] and anti-Lam antibodies and counted the 3D distances between CenpA signals and the nuclear lamina (Fig. 6b, Additional file 2: Fig. S9). The radial distribution of centromeres relative to the nuclear envelope (Additional file 8: Table S6) appears to be significantly different in these two cell types being shifted toward the nuclear interior in Kc167 cells (Fig. 6c). Therefore, in neurons centromeres are indeed closer to the nuclear lamina than in Kc167 cells.

## Discussion

### Active promoters are mostly located away from the nuclear lamina in *Drosophila*

Previously, *Drosophila* LADs were identified only in the Kc167 cell culture of embryonic origin [11]. In this study, we mapped LADs and inter-LADs in various organs/cell types, including the central brain, neurons, glia and the fat body from *Drosophila* third instar larvae. This allows us for the first time to reveal inter-LADs common to all cell types, which carry the vast majority of ubiquitously expressed gene promoters (Fig. 5a). Therefore, the permanent activity and/or the special chromatin type of ubiquitous promoters [40, 58] prevent them from contacting with the nuclear lamina in any cell type. We also found that, as in mammals [10, 53, 54], in *Drosophila* the active expression of some, but not all, tissue-specific gene promoters results in the loss of their association with the nuclear lamina (Fig. 5b, c). Taken together, this supports the long-standing paradigm that gene activity is poorly compatible with gene positioning at the nuclear lamina (reviewed in [59]), which also appears to be true for *Drosophila*.

### Tethering of HP1a/H3K9me2-enriched LADs to the nuclear lamina in *Drosophila* and mammals may proceed via similar mechanism

The mechanism of LADs tethering to the nuclear envelope remains elusive, especially in *Drosophila*. Hypothetically, two not mutually exclusive types of interactions may be responsible for LADs attachment to the nuclear lamina: Components of the lamina may recognize and specifically bind with either DNA motifs or inactive chromatin signatures. Both types of interactions were revealed in mammals. On one hand, the cKrox repressor, recognizing GAGA DNA motifs, in the complex with histone deacetylase HDAC3 and inner nuclear membrane protein Lap2β were shown to participate in the tethering of several LADs to the nuclear envelope in murine fibroblasts [60]. On the other hand, the chromatin marks such as H3K9me2/3 were found to be required for keeping LADs at the nuclear periphery in mammals [24, 61, 62]. The same histone marks are necessary for the localization of the heterochromatic transgene array at the nuclear periphery in the nematode [13, 63]. Our analysis indicates that the conserved LADs are remarkably less abundant in *Drosophila* (Fig. 2) compared to mammals, where they occupy 33% of non-repetitive genome [47]. Moreover, LADs are notably more variable in *Drosophila* than in mammals. Weak conservation of LADs among cell types with various expression patterns indicates that in *Drosophila*, unlike mammals, the chromatin features are dominant over specific nucleotide sequences in determination of chromatin positioning at the nuclear lamina. Nevertheless, our findings that LADs in the diverse differentiated cell types of *Drosophila* are HP1a-enriched (Fig. 3a, b), like LADs in mammals which are enriched with H3K9me2 [9, 23–25], point to an existence of a cognate mechanism responsible for heterochromatin attachment to the nuclear lamina in both cases.

### Different effects of HP1a on transcription of genes residing inside and outside LADs

In agreement with the previously formulated hypothesis [48, 64], we suggest that the increased HP1a binding in LADs may be necessary to prevent harmful transcriptional leakage of silent genes residing in the active chromatin environment. The HP1a enrichment on the male X chromosome may counteract the activity of dosage compensation complex. Several lines of evidence support this idea. Firstly, depletion of HP1a resulted in specific de-condensation of the X polytene chromosome in *Drosophila* males [65], as well as in the predominant male lethality [66], linking HP1a enrichment on the X chromosome (Fig. 3c, d) with a phenomenon of dosage compensation. Secondly, consistent with the previous results [11] and according to our data, silent or weakly expressed genes reside in LADs in all organs/cell types examined (Fig. 4a), and genes occupying the HP1a-enriched LADs are expressed even more weakly (Fig. 4b). Thirdly, artificial recruitment of HP1a to the promoter of a reporter gene, integrated at numerous sites in Kc167 cells, resulted in the threefold to fourfold repression of its transcription [67]. Finally, Lam depletion in the fat body of young flies caused a reduction in the level of HP1a accompanied by the derepression of a set of immune response genes [68], thus pointing to the collaboration of Lam and HP1a in the repression of genes unwanted in a particular tissue.

Several studies in *Drosophila* indicate that HP1a may be paradoxically involved in the activation of expression of a particular set of genes ([69–74], reviewed in [75]). Our results also indicate that outside LADs HP1a is bound to the subset of actively expressed euchromatic genes in Kc167 cells, as well as in the central brain, neurons, glia and fat body (Fig. 4c). However, the mode of HP1a binding to the actively expressed genes is likely different from its binding in LADs. Indeed, it was shown that HP1a interacts with the active RNA polymerase II and directly binds protein-coding gene transcripts [69]. In neurons, we found the increased association of HP1a with the promoters of actively expressed genes and rather uniform distribution of HP1a along the genes expressed at lower levels (Fig. 5c, right panel). Unfortunately, our analysis of microarray gene expression data from HP1a-depleted Kc167 cells [50] did not permit us to come to definite conclusions concerning the HP1a effects on the transcription of actively expressed genes. According to this analysis (Fig. 4d, Additional file 2: Fig S7D), HP1a may have either a neutral or the weak repressive effect on the transcription or RNA stability of these targets. However, it could not be excluded that strong side effects of HP1a depletion may mask its genuine action. Therefore, we propose that HP1a may enhance the repression of target genes, when bound in LADs, or affect transcription or transcript stability by an unknown mode (if any), when bound to the subset of actively expressed genes residing outside LADs.

### Maturation of heterochromatin in differentiated cells

Previously, the HP1a/H3K9me2-enriched chromatin in Kc167 cells was classified as the “GREEN” chromatin type [40]. These regions in Kc167 cells do not interact with the nuclear lamina (Fig. 3a) and mostly contain actively expressed genes (Fig. 4a). In the present study, we reveal in larval neurons and, less abundantly, in glia and the fat body the previously uncharacterized in *Drosophila* peripheral chromatin, which is bound by HP1a (Fig. 3a). Whether this is a novel heterochromatin type for *Drosophila* or a partial reposition of the “GREEN” chromatin type from nuclear interior to the nuclear lamina is currently unclear. We found that in the central brain the presence of TEs is significantly increased next to the HP1a-bound LADs, as compared to LADs without HP1a. Our findings point to the possibility that TEs may recruit HP1a as seeding points from which the HP1a spreads into the flanking regions. This idea is supported by the revealed spreading of H3K9me2/3-enriched chromatin on up to 20-kb distances from the TE insertion sites in *Drosophila* [76].

Besides binding of HP1a in LADs, we observe a general HP1a enrichment on the X chromosome, especially in males (Fig. 3c, d), which is in agreement with the previously reported data [48]. This enrichment may be a consequence of specific DNA motifs overrepresented on the X chromosome or different spatial proximity of the X chromosome and autosomes to the CHet compartments.

Our results are consistent with the analysis of Riddle et al. [64], who revealed the extended H3K9me2-enriched domains, occupied by HP1a, in the ChAs of *Drosophila* BG3 cell culture originated from the larval central nervous system [77], although similar domains were not detected in the embryonic Kc167 cells [64]. Taken together, these findings support the view that chromatin at the nuclear periphery becomes gradually HP1a/H3K9me2-enriched during terminal cell differentiation in *Drosophila* [78]. Interestingly, this is in contrast to mammals, where the Pc/H3K27me3- but not the H3K9me3-enriched domains expand in the tissues during development [79].

The overlap of LADs and HP1a domains in neurons occurs not only in the ChAs but also in the pericentromeric compartment. Such interactions are missing in Kc167 cells, at least at the distal pericentromeric regions probed by the microarrays used to generate the DamID profiles in these cells [40] (Fig. 3b). We cannot exclude, however, that satellite repeat regions, which located more proximal to centromeres and were not represented on the microarrays, may be bound with the nuclear lamina. Nevertheless, our immunostaining experiments revealed the closer positioning of centromeres to the nuclear envelope in neurons than in Kc167 cells (Fig. 6b, c) in line with the DamID results. These findings favor the model that HP1a-enriched pericentromeric compartments of individual chromosomes are attached to the nuclear lamina in neurons, but may be more randomly positioned around nucleolus in Kc167 cells ([80], reviewed in [81]). The relocalization of CHet compartments during differentiation is not unique to *Drosophila*. The LBR-dependent repositioning of pericentromeric regions from the nuclear lamina to the nuclear interior [82, 83] or vice versa [84] during differentiation of some mammalian cell types has previously been reported. Moreover, upon glial cell differentiation in mice, the pericentromeric regions were shown to increasingly associate with the nuclear periphery and repress active reporter genes artificially recruited to their proximity [85].

Recently, using targeted DamID approach [42], several factors linked to either repressive (Pc, HP1a, histone H1) or active (Brahma, RNA polymerase II) chromatin states in *Drosophila* neural stem cells and neurons were mapped genome wide [86]. This led to the conclusion that Pc-mediated repression does not play a significant role during neuronal differentiation. Instead, in neurons almost all key neural stem cell genes appear to be switched off via HP1a-mediated repression concomitant with the approximately twofold increase in HP1a genome coverage [86]. These conclusions are in agreement with the findings of the present study, namely, with the low variability of Pc domains in different organs/cell types (Fig. 2) and with the drastically higher HP1a genome occupancy in neurons as compared to embryonic Kc167 cells (Fig. 3d). Importantly, the mapping of LADs in various organs/cell types, described here, allowed to uncover that in various differentiated cell types, including neurons, the HP1a becomes enriched in the chromosomal regions associated with the nuclear lamina.

## Conclusions

Mapping of LADs in various organs/cell types of *Drosophila* third instar larvae shows that they are less conserved than LADs in mammals. In the terminally differentiated cells, such as neurons, *Drosophila* LADs become strongly occupied by HP1a, the reader of H3K9me2/3, which is in sharp contrast with Kc167 cells of embryonic origin. As LADs in mammals are enriched with H3K9me2/3, mechanisms of heterochromatin compaction and attachment to the nuclear lamina may be similar in *Drosophila* and mammals. Expression of genes located in LADs is generally very weak, and genes in the HP1-enriched LADs are expressed at the lowest level. Therefore, HP1a binding introduces an additional level of repression in LADs. The compartments of constitutive heterochromatin, revealed by centromere immunostaining, reside closer to the nuclear lamina in neurons than in Kc167 cells. Combined, these findings support the model that maturation of peripheral heterochromatin is required for the stronger repression of genes, which should not be expressed in the terminally differentiated cells.

## Methods

### Fly stocks and handling

Fly stocks were maintained under standard conditions at 25 °C. Transgenic fly lines bearing *Dam*^*4*-*HT*-*intein@L127C*^-*HP1* and *STOP#1*-*Dam*-*HP1* constructs were generated by φC31-mediated site-specific integration at the 51C site of the stock #24482 (the Bloomington Drosophila Stock Center) by BestGene company (http://www.thebestgene.com/). All DamID transgenic flies used in the study are available from the Bloomington Drosophila Stock Center under accession numbers #65429–65432 (intein system) and #65433–65436 (stop-cassette excision system). The *repo*-*FLP* stock [87] was kindly provided by Christian Klambt (Institut fur Neurobiologie, Universitat Munster, Munster, Germany). The *elav*-*GAL4* (#8760), *UAS*-*FLP* (#8208) and *UAS*-*RedStinger* (#8547) stocks were obtained from the Bloomington Drosophila Stock Center. To perform DamID in the fat body, intein excision was induced by 4-hydroxytamoxifen (4-HT; Sigma-Aldrich). For that, 4-HT was added to the fly food at a final concentration of 25 μM and then mated female flies were allowed to lay eggs on this food. Thus, larvae were exposed to 4-HT from hatching until they were collected in the third instar stage. To perform DamID in the central brain of third instar larvae, the spontaneous excision of intein (without 4-HT induction) [44] was utilized. The scheme of fly crossing for DamID profiling in neurons is presented in Additional file 1.

### DamID-seq

A few dozen central brains or fat bodies from wandering third instar male larvae or, in case of Repo-positive glia or Elav-positive neurons, from a mix of male and female larvae were manually dissected and collected as described previously [45]. Isolation of genomic DNA, amplification of Dam-methylated genomic fragments and their subsequent HTS were performed according to [45]. Eighteen cycles of PCR amplification (1 min at 94 °C, 1 min at 65 °C, 2 min at 68 °C) were applied for all DNA samples. HTS on Illumina HiSeq 2000 instrument was performed at the Genomics Core Facility of The Netherlands Cancer Institute and resulted in from ~ 25 to ~ 120 million 51-nt single-end reads per sample (Additional file 9: Table S7).

### Bioinformatic analysis of DamID-seq data

Sequencing reads from two biological replicates of Dam, Dam-Lam, Dam-HP1a or Dam-Pc samples for each organ/cell type were adapter clipped and uniquely mapped to the dm3/R5 genomic assembly by “bowtie2” [88]. Reads were counted by “HTSeq-count” software [89] in the 1-kb genomic bins. We employed equal size bins for mapping, as the HMM algorithm used to identify POI targets works “better” on bins of equal length than on the GATC–GATC fragments of various lengths. Bin size was determined empirically as a compromise between increased genome read coverage and decreased DamID resolution. Read counts were merged between replicates, as they were highly correlated (Additional file 2: Fig. S2). The resulting read counts of Dam or Dam-POI samples were converted to reads per million (RPM), and then, Dam-POI values were normalized to those of the Dam and log_2_ transformed. Since for each POI the dynamic range of log_2_-transformed profiles in different organs/cell types was rather different and we wanted to make cross-tissue comparisons, quantile normalization between organs/cell types was applied. We propose that variability in the dynamic range is caused by the different DamID approaches used (the induced/uninduced intein and stop-cassette excision systems) and has no biological relevance. This is supported by the high correlation between DamID profiles in the central brain and neurons, while the dynamic ranges of log_2_ profile in these organs/cell types were quite different. On the contrary, a substantially lower correlation was observed between the central brain and glial cells, which were just slightly different in the dynamic ranges of log_2_ profiles.

HP1a and Lam domain calling was performed with a two-state HMM algorithm (the scripts for DamID-seq analysis are available in the GitHub repository (https://github.com/foriin/DamID-seq). For determination of Pc domains, we applied three-state HMM, as for unknown reasons two-state HMM overestimated domain presence in the central brain (not shown). The domains for Lam, HP1a and Pc in Kc167 cells generated by DamID microarray approach [40] were retrieved from GSE22069. The median size of the Lam domains (Additional file 2: Fig. S3, [40]) appears to be smaller than was previously reported for Kc167 cells (~ 90 kb [11]) most probably due to different algorithms employed for domain calling. The actual domain sizes are likely much larger than provided in Additional file 2: Fig. S3 because HMM does not fill the gaps that originated over the bins that contained no mapped reads in the Dam profile. However, this underestimation of domain sizes does not distort the further analysis of domain/domain or gene/domain intersections which were computed in R as a ratio of genome coverage using the “GenomicRanges” package in Bioconductor [90]. To perform permutation analysis, we invoked “BEDTools” [91] in R to shuffle domains and TSSs (or genes) 10,000 times and then counted the number of TSSs (or genes) that intersected with domains or inter-domains.

### Bioinformatic analysis of RNA-seq and microarray expression data

RNA-seq data for the central larval brain and fat body were taken from GSE75835 [44], and for Kc167 cells—from GSE15596 [49]. RSEM software [92] was used for analysis, and transcripts per million (TPM) values were obtained as an output. Microarray expression data for control and HP1a-depleted Kc167 cells were retrieved from GSE18092 [50], converted back to the non-log_2_-transformed values (with *p *< 0.05) and averaged between replicates. Differentially expressed genes upon HP1a KD were determined using the “limma” R package [93]. Only genes with the cutoff for adjusted *p* values < 0.05 were used for further analysis.

### Generation of lists of ubiquitously or tissue-specifically expressed genes

If at least 3 (out of 4) present calls (i.e., values exceeding background) in each of 15 adult and larval *Drosophila* tissues/organs in the whole-transcriptome RNA-chip data (GSE7763 [51]) were found, the transcript was identified as being ubiquitously represented. If at least one spliced transcript variant of a gene was classified as ubiquitous, then the gene was identified as being ubiquitously expressed. As a result, 4377 ubiquitously expressed genes were revealed (Additional file 7: Table S5).

To generate the lists of tissue-specific genes expressed in neurons or glia, we employed RNA-seq data from larval neurons or glial cells (GSE71104 [52]). TPM values for two replicates were averaged. Protein-coding genes with TPM values ≥ 1 were considered as expressed. The tissue-specific gene lists were formed by the subtraction of ubiquitous genes from the lists of genes expressed in neurons or glia. Genes ranked by TPM values were separated into four groups according to their expression level (silent: TPM 0–1; low: TPM 1–10; medium: TPM 10–44 for neurons and 10–42 for glia; high: TPM > 44 for neurons and > 42 for glia) with an equal number of genes in each of the three last groups. For the plots in Fig. 5c, genes were oriented in the 5′ → 3′ direction starting from their promoters (distal TSSs) and medians of non-quantile normalized log_2_(Dam-POI/Dam) values across seven genomic bins centered at gene promoters (three bins upstream and three bins downstream from the zero bin) were calculated. Only genes overlapped with the corresponding domain type by their bodies and only the upstream bins not overlapped with other genes and the downstream bins carrying the corresponding gene were taken for the analysis.

### Analysis of distribution of TEs in the genome

Genomic positions for 3183 TE insertions in the *Drosophila* dm3/R5 genome assembly (within the ChAs lacking the distal pericentromeric regions: i.e., within 1–22,300 kb of X chromosome, 1–22,000 kb of 2L chromosome, 1600–21,147 kb of 2R chromosome, 1–22,900 kb of 3L chromosome, 1–27,900 kb of 3R chromosome, according to Riddle et al. [64]) were downloaded from the FlyBase ftp site (ftp://ftp.flybase.net/releases/FB2014_03/dmel_r5.57/gff/dmel-all-r5.57.gff.gz). To analyze whether there is any preference in the TE localization in the vicinity of LADs bound or unbound with the HP1a, we estimated the observed to expected number of insertion events after random reshuffling of TEs and domains for 10^4^ times (i.e., in the permutation test). As we considered only uniquely mapped reads during DamID-seq analysis, the TE sequences were mostly excluded from the identified LADs and HP1a domains. Therefore, the observed number of insertion events was counted as a number of overlapping events between positions of TEs, extended by 0.5 kb from their ends, and the domains.

### Statistical analysis

For *p* value estimation, the Mann–Whitney (M–W) *U* test was used for comparison of two sample distributions. *p* values for occasional gene/domain or domain/domain overlapping were estimated by permutation test with 10,000 permutations.

### Cell culture

Kc167 cells obtained from Drosophila Genomics Resource Center were grown in Schneider’s *Drosophila* medium (Gibco) supplemented with 10% heat-inactivated FBS (Gibco), 50 units/ml penicillin and 50 µg/ml streptomycin.

### Immunostaining

Immunostaining was performed as previously described in [94] with some modifications. Kc167 cells in the growth phase were collected and rinsed two times in PBS. Central brains from third instar larvae were manually isolated in PBT (PBS containing 0.01% Tween-20) on ice and then rinsed in PBS. Cells or brains were fixed in 4% formaldehyde (in PBT) for 25 min at room temperature. Fixation was stopped by incubation with 0.25 M glycine (Sigma-Aldrich) for 5 min. Then, cells or brains were washed in PBS three times for 10 min at room temperature, permeabilized with PBTX (PBS with 0.1% Tween-20, 0.3% Triton X-100) for 10 min, blocked with PBTX containing 3% normal goat serum (NGS, Invitrogen) at room temperature for 1 h (cells) or for 3 h (brains), incubated with primary antibody in PBTX containing 3% NGS for 3 h (cells) or for 7 h (brains) at room temperature, or overnight at 4 °C, washed in PBTX three times for 10 min at room temperature, incubated with secondary antibodies (1:1000) in PBTX containing 3% NGS for 3 h (cells) or for 7 h (brains) at room temperature, or overnight at 4 °C, and then washed in PBTX three times for 10 min at room temperature in a dark chamber. Coverslips were mounted with a drop of SlowFade Gold Antifade reagent (Invitrogen) containing DAPI. As the primary, rabbit polyclonal anti-HP1a (1:500, Covance #PRB-291C), mouse monoclonal anti-Lam (ADL84, 1:500 [95]), or chicken polyclonal anti-CenpA (CID, 1:600, [57]) antibodies were used. As the secondary, Alexa Fluor 488-conjugated goat anti-rabbit IgG (Invitrogen), Alexa Fluor 488-conjugated, Alexa Fluor 633-conjugated goat anti-mouse IgG (Invitrogen), or Alexa Fluor 633-conjugated goat anti-chicken IgG (Invitrogen) antibodies were used.

### Measuring distances from centromeres to the nuclear lamina

Three-dimensional image stacks were recorded with a confocal LSM 510 Meta laser scanning microscope (Zeiss). Optical sections were captured at 0.4–0.6-μm intervals along the *Z*-axis. Images were processed and analyzed using IMARIS 7.4.2 software (Bitplane AG) with a blind experimental setup. Images were thresholded to eliminate hybridization and immunostaining background effects. The distances between signals and the nuclear envelope were counted as previously described [16]. Briefly, nuclear lamina stained by anti-Lam antibodies was manually outlined by its middle in each plane of the Z-stack, before automatic reconstruction of the nuclear surface by IMARIS. One measurement point was positioned in the optical section with the brightest CenpA signal, at its visually determined center, and another one was placed on the reconstructed nuclear surface at the point of its earliest intersection with the progressively growing sphere from the first measurement point. The distance between the measurement points (the shortest distance between the center of CenpA signal and the middle of nuclear lamina) was measured for each nucleus. Data were obtained in two independent experiments for 50–60 nuclei per experiment (Additional file 8: Table S6). Distances were normalized on the nuclei radii, and radial-normalized distances in neurons and Kc167 cells were compared.

## Additional files

**Additional file 1.** Scheme of fly crossing for DamID in neurons.

**Additional file 2.** Figures S1–S9.

**Additional file 3. Table S1:** HP1a, Lam and Pc domain coordinates in the central brain, neurons, glia and fat body.

**Additional file 4. Table S2:** The percentage of intersected domain length from total POI domain length (where POI is HP1a, Lam or Pc) in the central brain, neurons, glia, fat body and Kc167 cells separately for ChAs, CHet, 4th chromosome and non-repetitive part of X chromosome.

**Additional file 5. Table S3:** Expression of genes according to the RNA-seq data from [44, 49] with the indication of promoter location within HP1a, Lam or Pc domains in the central brain, fat body and Kc167 cells.

**Additional file 6. Table S4:** Differentially expressed gene list upon HP1a KD in Kc167 cells with the indication of intersection of gene bodies with HP1a, Lam or Pc domains. Microarray expression data were from [50].

**Additional file 7. Table S5:** List of ubiquitously expressed genes based on microarray expression data from [51] with the indication of promoter location within the conserved HP1a, Lam and Pc domains or within the conserved inter-domains.

**Additional file 8. Table S6:** Distances from the CenpA signals to the nuclear lamina in Elav-positive neurons and Kc167 cells.

**Additional file 9. Table S7:** HTS raw data parameters.

## Abbreviations

4-HT: 4-hydroxytamoxifen
ChAs: chromosome arms
CHet: constitutive heterochromatin (the 2LHet, 2RHet, 3LHet, 3RHet, XHet chromosome regions of dm3/R5 genome assembly)
H3K27me3: trimethylated Lys27 of histone H3
H3K9me2: dimethylated Lys9 of histone H3
HMM: hidden Markov model
HP1a: heterochromatin protein 1a
HTS: high-throughput sequencing
KD: knockdown
LADs: lamina-associated chromosomal domains
Lam: lamin Dm0
M–W *U* test: Mann–Whitney *U* test
NGS: normal goat serum
Pc: Polycomb
PcG: Polycomb group
PCR: polymerase chain reaction
POIs: proteins of interest
RPM: reads per million
TEs: transposable elements
TPM: transcripts per million
TSS: transcription start site
