# Supplementary material for: The large fraction of heterochromatin in Drosophila neurons is bound by both B-type lamin and HP1a
Source: Epigenetics Chromatin. 2018 Nov 1;11:65. doi: 10.1186/s13072-018-0235-8 (PMC6211408; doi:10.1186/s13072-018-0235-8)
Supplement: Supplementary file 1 — Additional file 1. Scheme of fly crossing for DamID in neurons. [file 13072_2018_235_MOESM1_ESM.pdf]

Scheme of fly crossing for DamID in neurons (exemplified by Dam and Dam-Lam):

1a) ♀♀ *M{hsp70P.min-s1-Dam}ZH51C-M2* × ♂♂ *CyO/nub*^{1} *b*^{1} *sna*^{Sco} *lt*^{1} *stw*^{3}; *MKRS/TM6B, Tb*^{1} → ♂♂ *M{hsp70P.min-s1-Dam}ZH51C-M2/nub*^{1} *b*^{1} *sna*^{Sco} *lt*^{1} *stw*^{3}; *+/TM6B, Tb*^{1}

1b) ♀♀ *M{hsp70P.min-s1-Dam-LAM}ZH51C-M1* × ♂♂ *CyO/nub*^{1} *b*^{1} *sna*^{Sco} *lt*^{1} *stw*^{3}; *MKRS/TM6B, Tb*^{1} → ♂♂ *M{hsp70P.min-s1-Dam-LAM}ZH51C-M1/nub*^{1} *b*^{1} *sna*^{Sco} *lt*^{1} *stw*^{3}; *+/TM6B, Tb*^{1}

1c) ♀♀ *P(GAL4-elav.L)3* × ♂♂ *Sp/CyO*; *Sb*^{1}*/TM6, Ubx* → ♀♀ *+/CyO*; *P(GAL4-elav.L)3/TM6, Ubx*

2ac) ♀♀ *+/CyO*; *P(GAL4-elav.L)3/TM6, Ubx* × ♂♂ *M{hsp70P.min-s1-Dam}ZH51C-M2/nub*^{1} *b*^{1} *sna*^{Sco} *lt*^{1} *stw*^{3}; *+/TM6B, Tb*^{1} → ♂♀ *M{hsp70P.min-s1-Dam}ZH51C-M2/CyO*; *P(GAL4-elav.L)3/TM6B, Tb*^{1}

2bc) ♀♀ *+/CyO; P(GAL4-elav.L)3/TM6, Ubx* × ♂♂ *M{hsp70P.min-s1-Dam-LAM}ZH51C-M1/nub*^{1} *b*^{1} *sna*^{Sco} *lt*^{1} *stw*^{3}; *+/TM6B, Tb*^{1} → ♂♀ *M{hsp70P.min-s1-Dam-LAM}ZH51C-M1/CyO*; *P(GAL4-elav.L)3/TM6B, Tb*^{1}

3ac) ♀♀ *M{hsp70P.min-s1-Dam}ZH51C-M2/CyO*; *P(GAL4-elav.L)3/TM6B, Tb*^{1} × ♂♂ *M{hsp70P.min-s1-Dam}ZH51C-M2/CyO*; *P(GAL4-elav.L)3/TM6B, Tb*^{1} → ♂♀ *M{hsp70P.min-s1-Dam}ZH51C-M2*; *P(GAL4-elav.L)3*

3bc) ♀♀ *M{hsp70P.min-s1-Dam-LAM}ZH51C-M1/CyO*; *P(GAL4-elav.L)3/TM6B, Tb*^{1} × ♂♂ *M{hsp70P.min-s1-Dam-LAM}ZH51C-M1/CyO*; *P(GAL4-elav.L)3/TM6B, Tb*^{1} → ♂♀ *M{hsp70P.min-s1-Dam-LAM}ZH51C-M1*; *P(GAL4-elav.L)3*

4ac) ♀♀ *P{UAS-FLP.Exel}1, y*^{1} *w*^{1118} × ♂♂ *M{hsp70P.min-s1-Dam}ZH51C-M2*; *P(GAL4-elav.L)3* → isolation of central brains from ♂♀ *P{UAS-FLP.Exel}1, y*^{1} *w*^{1118}/+; *M{hsp70P.min-s1-Dam}ZH51C-M2*/+; *P(GAL4-elav.L)3*/+ of third instar larvae.

4bc) ♀♀ *P{UAS-FLP.Exel}1, y*^{1} *w*^{1118} × ♂♂ *M{hsp70P.min-s1-Dam-LAM}ZH51C-M1*; *P(GAL4-elav.L)3* → isolation of central brains from ♂♀ *P{UAS-FLP.Exel}1, y*^{1} *w*^{1118}/+; *M{hsp70P.min-s1-Dam-LAM}ZH51C-M4*/+; *P(GAL4-elav.L)3*/+ of third instar larvae.
