# Supplementary material for: The large fraction of heterochromatin in Drosophila neurons is bound by both B-type lamin and HP1a
Source: Epigenetics Chromatin. 2018 Nov 1;11:65. doi: 10.1186/s13072-018-0235-8 (PMC6211408; doi:10.1186/s13072-018-0235-8)
Supplement: Supplementary file 2 — Additional file 2. Figures S1–S9. [file 13072_2018_235_MOESM2_ESM.pdf]

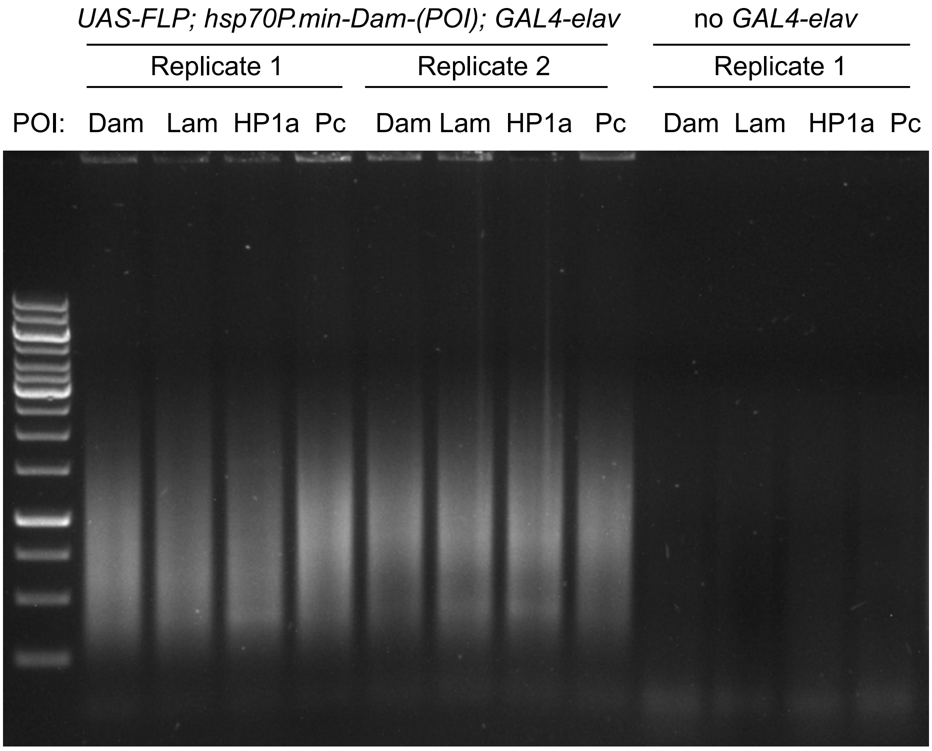

Fig. S1. High specificity of the DamID procedure in Elav-positive neurons. Gel-electrophoresis of PCR-amplified methylated genomic DNA from indicated genotypes are shown. Drastically less PCR-amplified DNA is seen in the samples where the GAL4 driver was omitted and, as a result, the stop-cassette was not removed in the constructs (e.g. no *GAL4-elav*).

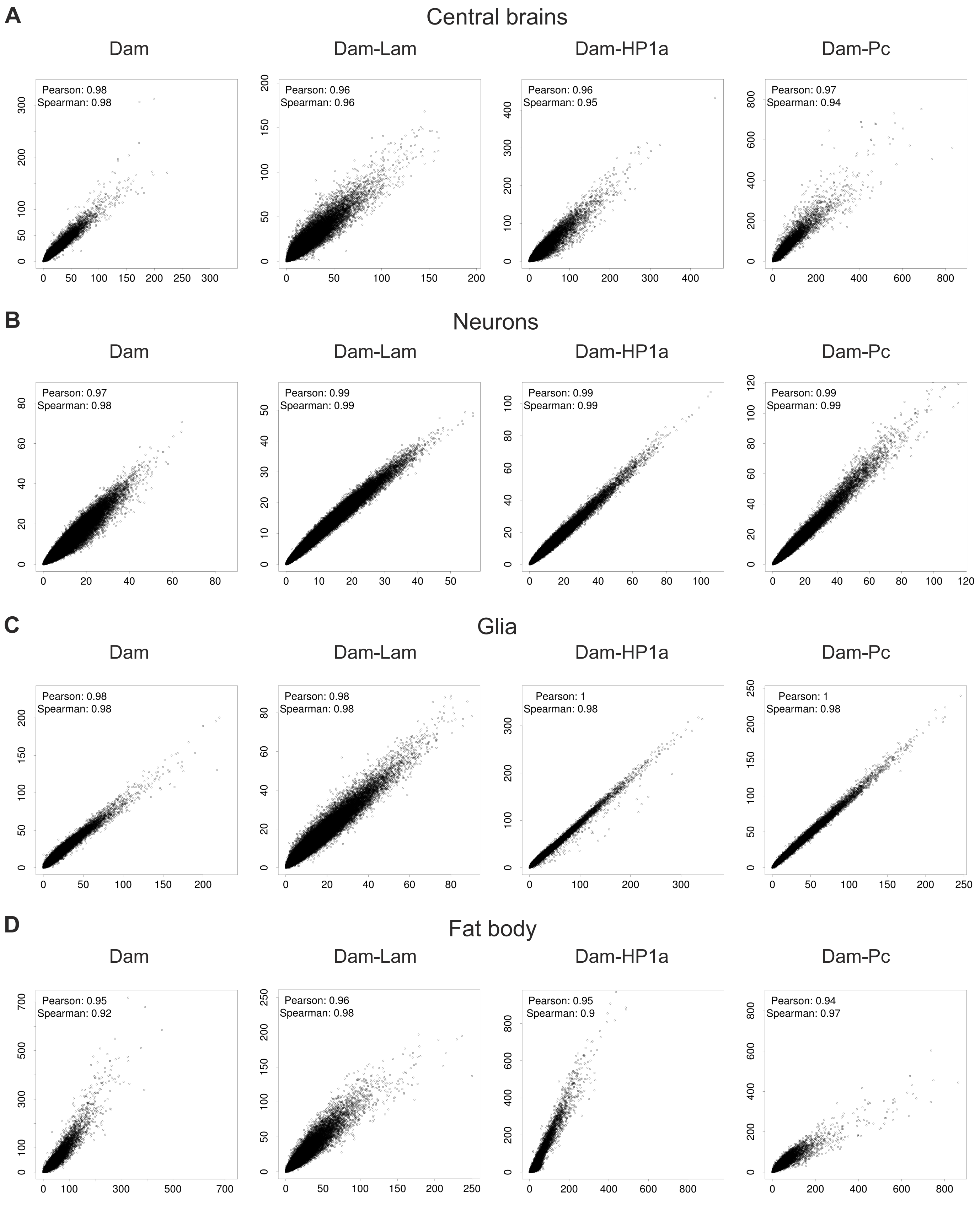

Fig. S2. Reproducibility of DamID replicates (1st *vs* 2nd) for the central brain (**A**), Elav-positive neurons (**B**), Repo-positive glia (**C**), or fat body (**D**). RPM values for replicates 1 and 2 are shown along *X* and *Y* axis, respectively.

A

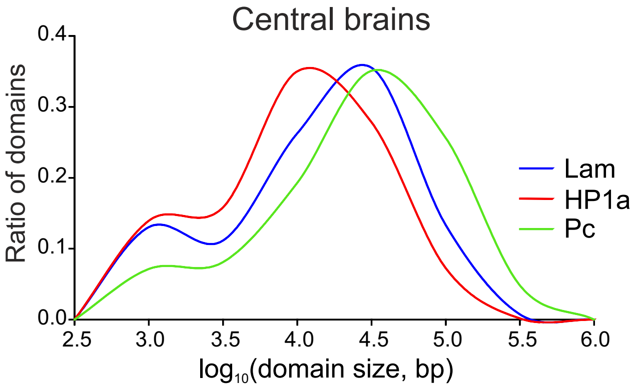

	Number of domains	Median domain size, bp
HP1a	3250	7000
Lam	3117	10000
Pc	519	17000

B

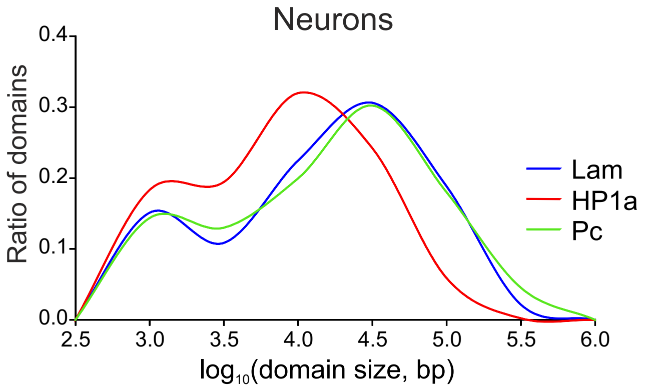

	Number of domains	Median domain size, bp
HP1a	3744	5000
Lam	3291	11000
Pc	662	11000

C

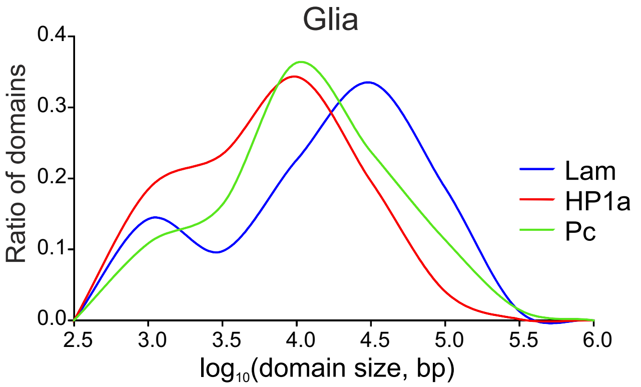

	Number of domains	Median domain size, bp
HP1a	3451	4000
Lam	3675	12000
Pc	1006	7000

D

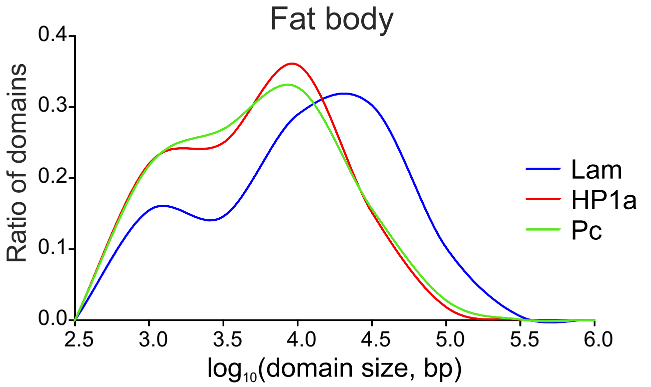

	Number of domains	Median domain size, bp
HP1a	3676	4000
Lam	4256	8000
Pc	2654	4000

E

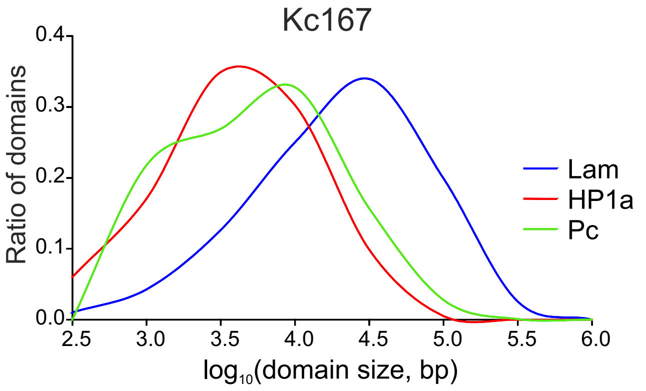

	Number of domains	Median domain size, bp
HP1a	1753	2355
Lam	2460	12700
Pc	2027	8267

Fig. S3. Domain distribution by size in the central brain (**A**), Elav-positive neurons (**B**), Repo-positive glia (**C**), fat body (**D**), or Kc167 cells (**E**).

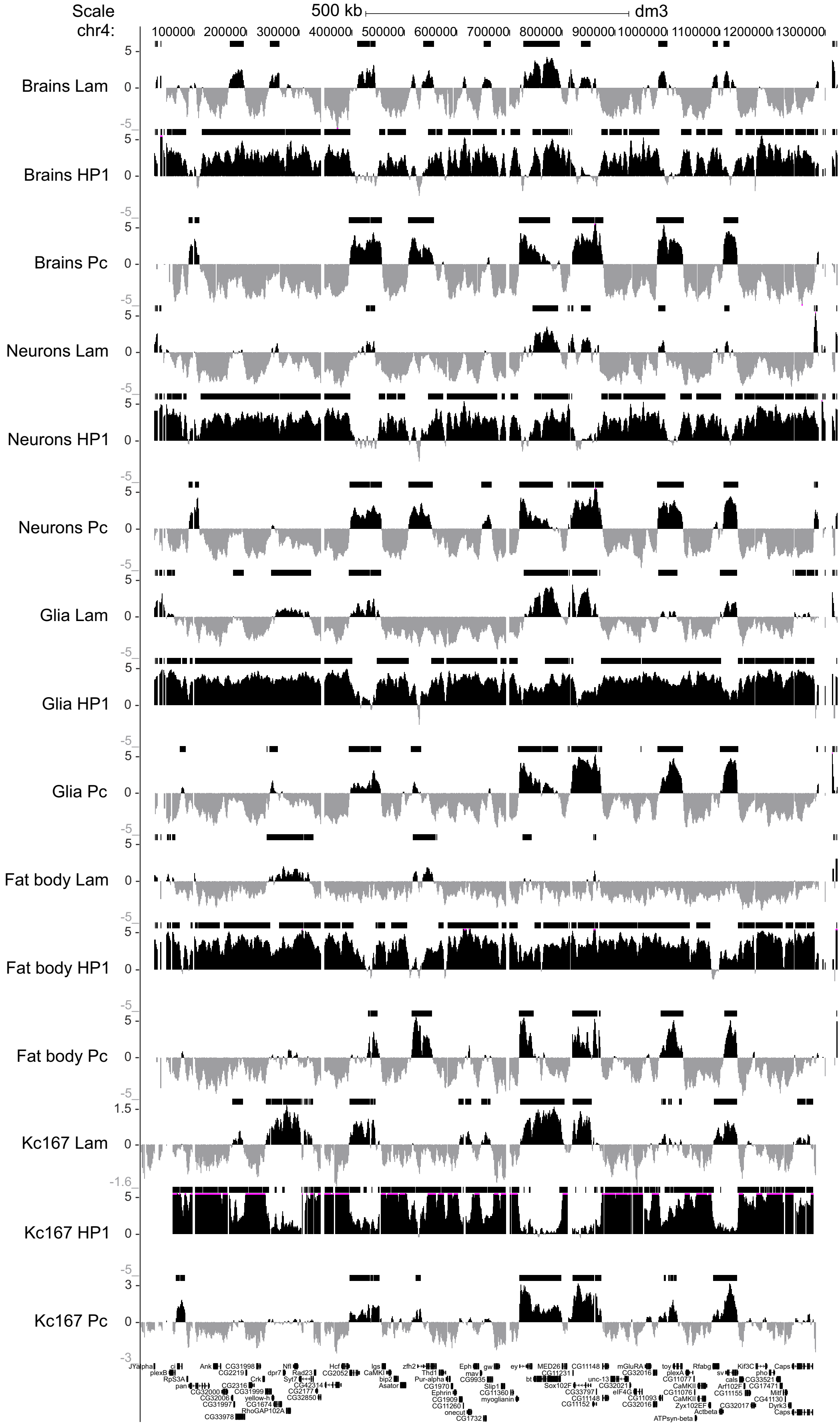

Fig. S4. Screenshot from UCSC genome browser showing $\log_2$(Dam-POI/Dam) profiles (where POI is Lam, HP1a or Pc) and HMM-determined domains (black rectangles over profiles) for the whole 4th chromosome region, represented in the *Drosophila* dm3/R5 genome assembly, for the central brain, neurons, glia, fat body and Kc167 cells. Data for Kc167 cells were taken from [11,40].

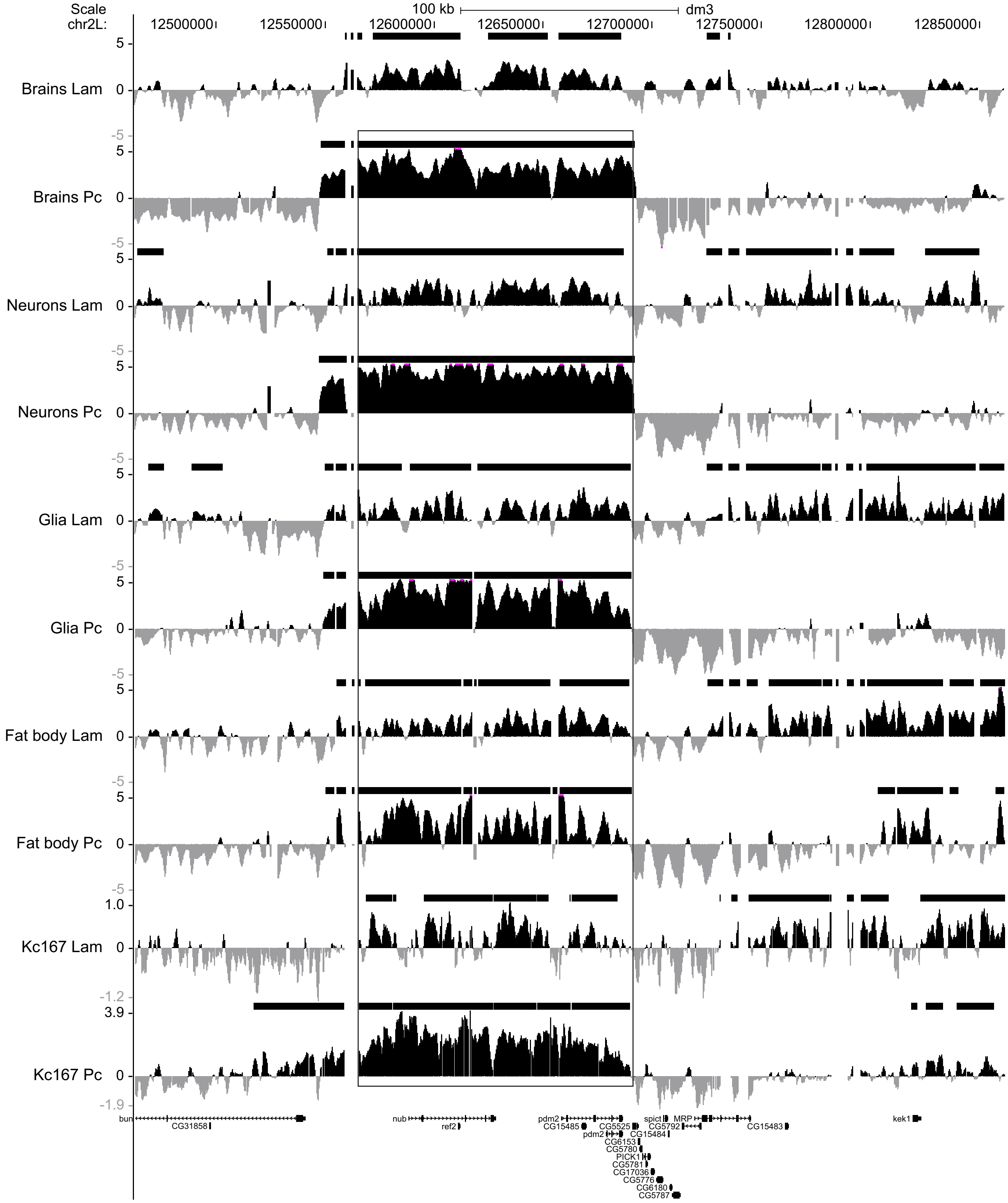

Fig. S5. Example of conserved Pc domains overlapped with LADs. Screenshot from UCSC genome browser showing log_2(Dam-POI/Dam) profiles (where POI is Lam or Pc) and HMM-determined domains (black rectangles over profiles) for the representative 2L region in the central brain, neurons, glia, fat body and Kc167 cells. Data for Kc167 cells were taken from [11,40]. Example of conserved Pc domain is outlined by a black rectangle.

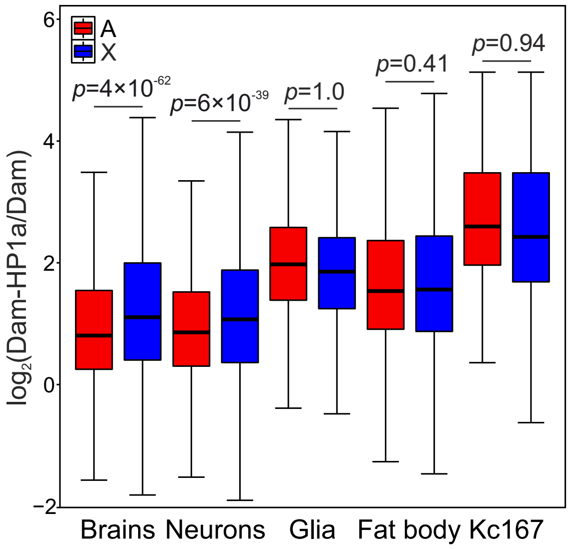

Fig. S6. $\log_2$(Dam-HP1a/Dam) values are higher on the X chromosome than on autosomes for the HP1a domains overlapped with LADs in the central brain and in neurons. Box plots showing distributions of $\log_2$(Dam-HP1a/Dam) values in the HP1a domains overlapped with LADs in the non-repetitive parts of X chromosome (blue) and autosomes (red) in the male larval central brain, male larval fat body, neurons or glial cells from mixed sex larvae, and in female Kc167 cells. For this type of analysis, raw DamID-seq data for HP1a in Kc167 cells were taken from GSE83713 [67], mapped on the 1-kb genomic bins and quantile normalized. Only ChA parts which, according to Riddle et al. [64], were within 1 - 22300 kb for X chromosome, 1 - 22000 kb for 2L, 1600 - 21147 kb for 2R, 1 - 22900 kb for 3L, 1 - 27900 kb for 3R of *Drosophila* dm3/R5 genome assembly, were taken for analysis. M-W U-test was used for pairwise comparison of distributions on the X chromosome *vs* autosomes.

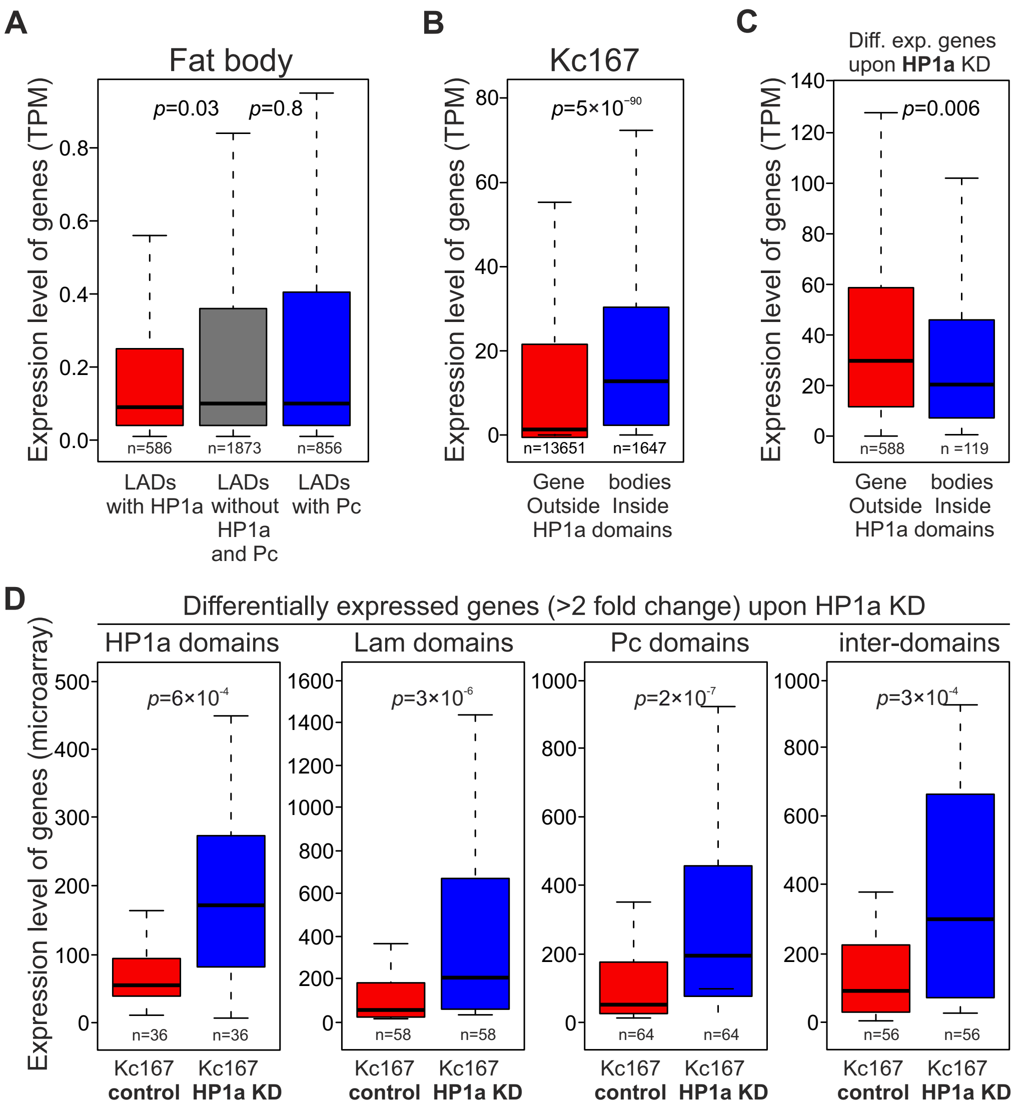

Fig. S7. Effects of HP1a on transcription. (**A**) Box plots (non-outlier range) showing expression of genes (in TPM) in the fat body whose promoters (distal TSSs) are located in LADs (left to right), overlapped with HP1a domains (red), non-overlapped with either HP1a or Pc domains (grey), or overlapped with Pc domains (blue). RNA-seq data for fat bodies were taken from GSE75835 [44]. Zero TMP values were removed from the analysis. (**B**) Box plots (non-outlier range) showing expression of genes (in TPM) overlapped (blue) or non-overlapped (red) by their bodies with HP1a domains in Kc167 cells. RNA-seq data for Kc167 cells were taken from GSE15596 [49]. (**C**) Box plots (non-outlier range) showing expression of genes in Kc167 cells (in TPM) for the differentially expressed genes upon HP1a KD, overlapped (blue) or non-overlapped (red) with HP1a domains by their bodies. RNA-seq data for Kc167 cells were taken from GSE15596 [49]. (**D**) Box plots (non-outlier range) showing expression levels for the differentially expressed genes (with more than two-fold expression change), overlapped by their bodies with HP1a, Lam or Pc domains, or not overlapped with any domain type, in the control Kc167 cells (red) or upon HP1a KD in Kc167 cells (blue). RNA-expression microarray data for analysis were taken from GSE18092 [50]. In (**A**–**D**) M-W U-test was used for pairwise comparison of distributions.

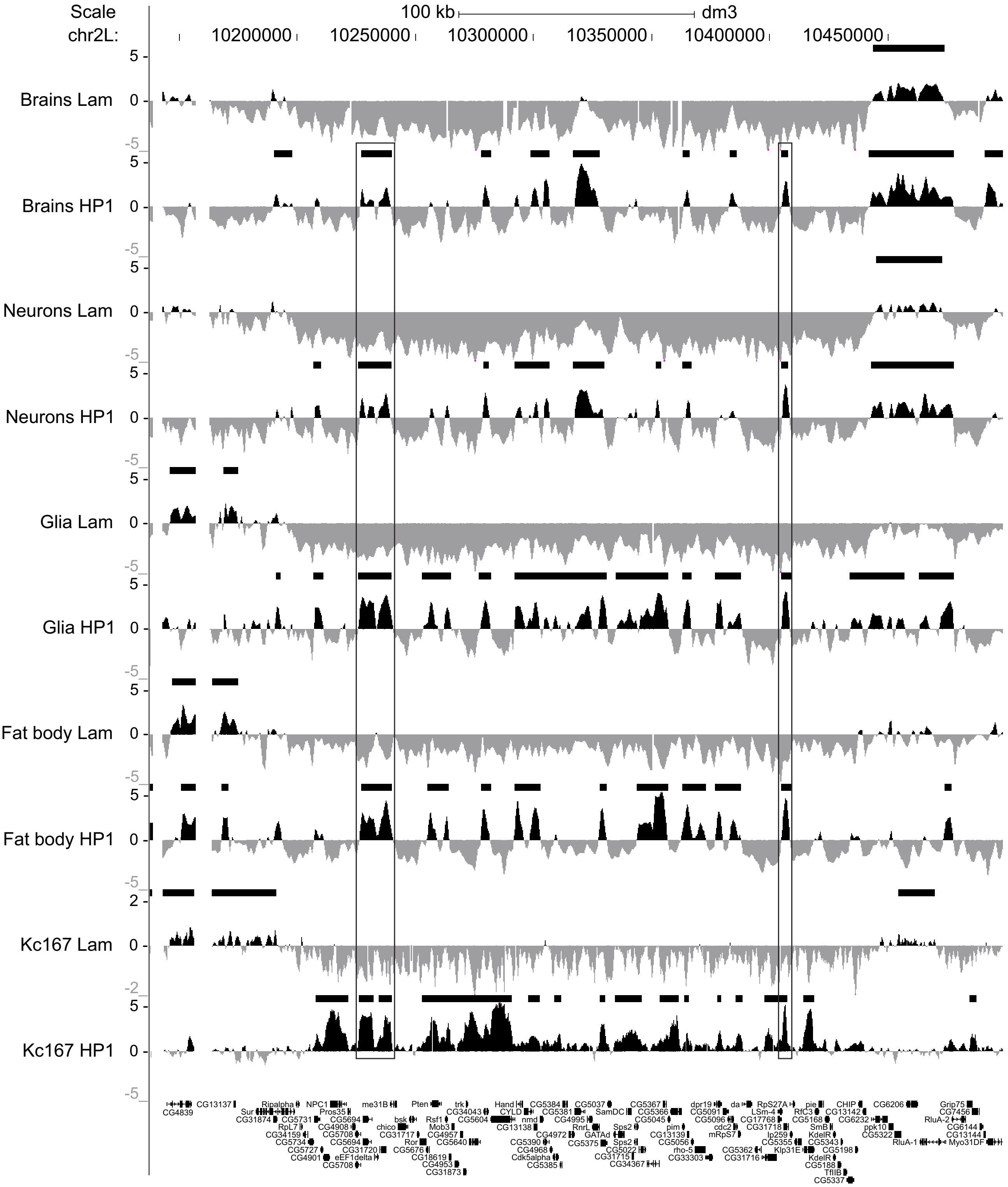

Fig. S8. HP1a binding with actively expressed genes is partially conserved in various cell types. Screenshot from UCSC genome browser showing $\log_2$(Dam-POI/Dam) profiles (where POI is Lam or HP1a) and HMM-determined domains (black rectangles over profiles) for the representative 2L region with the conserved HP1a domains in the central brain, neurons, glia, fat body and Kc167 cells. Data for Kc167 cells were taken from [11,40]. Examples of conserved HP1a domains, carrying actively expressed genes, are outlined by black rectangles.

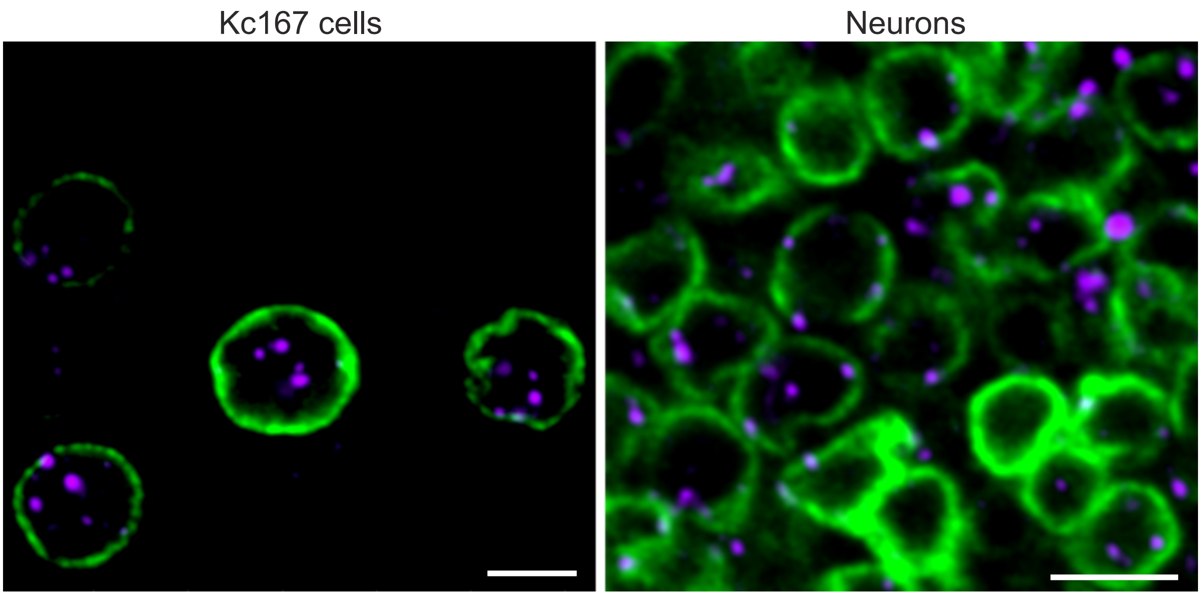

Fig. S9. Centromeres are located more closely to the nuclear envelope in neurons than in Kc167 cells. Immunostaining of Kc167 cells (left panel) or neurons (right panel) with anti-CenpA (violet) and anti-Lam (green) antibodies. Scale bars 5 μm.
